# Early Warning Signals for Critical Transitions: A Generalized Modeling Approach

**DOI:** 10.1371/journal.pcbi.1002360

**Published:** 2012-02-02

**Authors:** Steven J. Lade, Thilo Gross

**Affiliations:** Max Planck Institute for the Physics of Complex Systems, Dresden, Germany; University of Michigan and Howard Hughes Med. Inst., United States of America

## Abstract

Critical transitions are sudden, often irreversible, changes that can occur in a large variety of complex systems; signals that warn of critical transitions are therefore highly desirable. We propose a new method for early warning signals that integrates multiple sources of information and data about the system through the framework of a generalized model. We demonstrate our proposed approach through several examples, including a previously published fisheries model. We regard our method as complementary to existing early warning signals, taking an approach of intermediate complexity between model-free approaches and fully parameterized simulations. One potential advantage of our approach is that, under appropriate conditions, it may reduce the amount of time series data required for a robust early warning signal.

## Author Summary

Fisheries, coral reefs, productive farmland, planetary climate, neural activity in the brain, and financial markets are all complex systems that can be susceptible to sudden changes leading to drastic re-organization or collapse. A variety of signals based on analysis of time-series data have been proposed that would provide warning of these so-called critical transitions. We propose a new method for calculating early warning signals that is complementary to existing approaches. The key step is to incorporate other available information about the system through the framework of a so-called generalized model. Our new approach may help to anticipate future catastrophic regime shifts in nature and society, allowing humankind to avert or to mitigate the consequences of the impending change.

## Introduction

Critical transitions are sudden, long-term changes in complex systems that occur when a threshold is crossed [1]. Many systems are known to be at risk of such transitions, including systems in ecology [2], climate research [3], economics [4], sociology [5] and human physiology [6]. Examples of critical transitions in ecology include shifts in food web composition in shallow lakes [7], degradation of coral reefs [8], degradation of managed rangelands [9], and desertification [10].

Warning signals for impending critical transitions are highly desirable, because it is often difficult to revert a system to the previous state once a critical transition has occurred [2], [11]. If an accurate mathematical model of the system is available then critical transitions can be predicted straight-forwardly, either by numerical simulation or by direct computation of the dynamical thresholds. For real world complex systems, however, sufficiently accurate models are in general not available, and predictions based on models of limited accuracy face substantial difficulties [12]. Recent research has therefore focused on model-free approaches that extract warning signals from observed time series [13]. Two of the most widely used approaches are increasing variance [14] and autocorrelation [15], both of which are caused by critical slowing down [16]. Other approaches consider warning signals based on skewness [17], flickering [18] and spatial correlation [19].

One strategy for improving the quality of an early warning signal, which to our knowledge has not been explored, is to utilize other information that may be available. This other information may take the form of other time series data, for example in ecological applications birth rates as well as population sizes, or additional knowledge about the system, such as that the top-predator mortality is likely to be linear. This highlights the need for intermediate approaches, which can efficiently incorporate available information without requiring a fully specified mathematical model.

In the present Letter, we propose an approach for the prediction of critical transitions based on the framework of generalized modeling [20], [21]. The approach allows available information to be used, subject to certain limitations on the quality and availability of the information. Our results indicate that in the cases considered here, the approach can reduce the amount of time series data required or increase the quality of the prediction. We demonstrate the applicability of the proposed approach by considering a simple one-population model, a previously studied fisheries model and a tri-trophic food chain.

## Methods

### Generalized modeling

Suppose that a system has been identified as being at risk of a critical transition. Even if very little specific information is available, the dynamics can generally still be captured by a so-called generalized model [20]. Such a model captures the structure of the system, without restricting it to specific functional forms.

**Figure 1:**
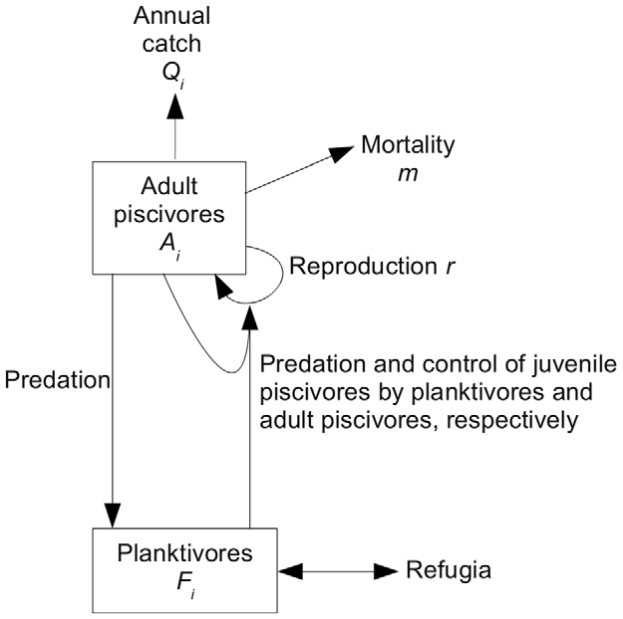
Schematic of the fishery knowledge that was incorporated into the generalized model.

To formulate a generalized model we first identify important system variables (say, abundance or biomass of the populations in the system) and processes (for example, birth, death, or predation). As a first step, the generalized model can then be sketched in graphical form, such as in Fig. 1 below. This graphical representation is sometimes called a causal loop diagram [22].

To obtain a mathematical representation of the model we write a dynamical equation for each variable 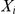. In these equations we represent the processes by general functions. For instance we can model a single population 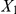 subject to gains 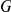 and losses 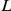 by an ordinary differential equation
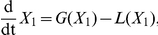
or as a discrete-time map
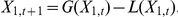
Note, that in contrast to conventional models, we do not attempt to describe the processes G and L by specific functional forms. Instead, we use unspecified functions 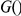 and 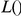 as formal placeholders for the (unknown) relationships realized in the real system.

### Calculation of early warning signal

We assume that before the critical transition, the system can be considered in equilibrium. We emphasize that this does not require the system to be completely static or closed in a thermodynamic sense, but that, on the chosen macroscopic level of description, the system can be considered at rest. For example, the system may be undergoing stochastic fluctuations of a fixed distribution around a stable fixed point. Additionally, the system is subject to a slowly changing external parameter that puts it at risk of undergoing a critical transition. The system is therefore at equilibrium only on a certain timescale. In the following we refer to this timescale as the *fast timescale*, while the dynamics of the whole system, including the slow change of the external parameter, takes place on the *slow timescale*.

Using the definitions above critical transitions can be linked to instabilities (bifurcations) of the fast subsystem [23]. For detecting these instabilities we construct the Jacobian matrix, a local linearization of the system around the steady state [24]. A system of ordinary differential equations (ODEs) is dynamically stable if all eigenvalues of the Jacobian have negative real parts, whereas a discrete time map is stable if all eigenvalues have an absolute value less than one. Critical transitions are thus signified by a change in the external parameter causing at least one of the eigenvalues to cross the imaginary axis (ODE) or a unit circle around the origin (map).

To warn of impending critical transitions we monitor the eigenvalues of the Jacobian of the fast subsystem, which usually change slowly in time. A warning is raised if at least one of the eigenvalues shows a clear trend toward the stability boundary (for ODEs, zero real part; for maps, absolute value of one). The Jacobian itself can be computed directly from the generalized model, but will contain unknown terms reflecting our ignorance of the precise functional forms in the model. Previous publications [20] have shown that these unknowns can be treated as well-defined parameters with clear ecological interpretations. In the present applications we estimate the unknowns in the Jacobian matrix from short segments of time series data. Thereby, a pseudo-continuous monitoring of the eigenvalues of the fast subsystem is possible.

The generalized model that is constructed should reflect existing knowledge about the structure of the system. It should contain terms that represent relevant and observable processes (or relevant processes whose magnitudes can be deduced from other processes, as we will see below). The generalized model should also have a structure that permits bifurcations that are relevant for the system; if not, the generalized model cannot be used to anticipate those bifurcations.

We note that with given time series data estimating the generalized model parameters is simpler than estimating the entries of the Jacobian matrix directly, because the generalized model already incorporates structural information about the system. Further, many of the parameters in the generalized model may already be available in a given application, because they refer to well-studied properties of the species, such as natural life expectancy or metabolic rate.

## Results

We applied the proposed approach to three case studies, focusing on a generic population with an Allee effect, a fisheries example, and a tri-trophic food chain.

### Simulation with Allee effect

Allee effects, that is, positive relationships between per-capita growth rate and population size, are postulated in many populations and have been conclusively demonstrated in some [25]. A population with an Allee effect can suddenly transition from a stable, non-zero population size to unconditional extinction [26].

We supposed that an early warning signal was desired for a population in which a slowly increasing death rate (for example the spread of a new disease, the appearance of a new predator, or habitat destruction) was pushing the population towards a critical transition associated with an Allee effect. We assumed that regular observations of the population size and birth rate were available.

Accordingly, we constructed the generalized model
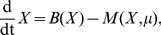
(1)where 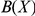 and 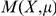 are the birth and death rates of the population, respectively, and 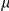 represents the external factor pushing the system towards the critical transition. We refer to the population and birth rate observations as 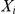 and 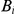, taken at times 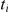, 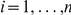. (Observations of the death rate would also be acceptable in place of the birth rate.)

From the generalized model of Eq. (1), we constructed the Jacobian (in this 1-D system, also the eigenvalue) of the system
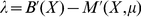
(2)near its steady state, where the prime denotes the derivative with respect to 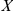. To calculate the changing values of the eigenvalue 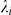 as the external parameter changes, we need to estimate the gradients 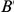 and 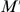 from our time series observations of 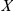 and 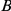.

We calculated 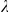 as follows. Since the birth rate 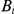 and the population 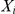 have been directly observed, 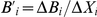 could therefore be computed immediately, where we use the notation 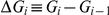. (These one-sided derivative estimators involve a loss in accuracy but allow the eigenvalues to be estimated at the most recent observation time, which is important when attempting to predict an imminent transition.) A discretization of Eq. (1) gives 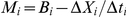. We cannot calculate 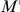 in the same way as 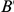, because 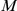 also depends on 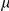. Instead, we make one additional assumption: That the mortality 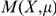 is linear in 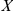 (although the coefficient of this linearity may change with 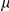). Then we can estimate 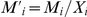. (Suppose 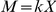. Then 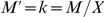.) Finally, the eigenvalue 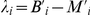.

**Figure 2:**
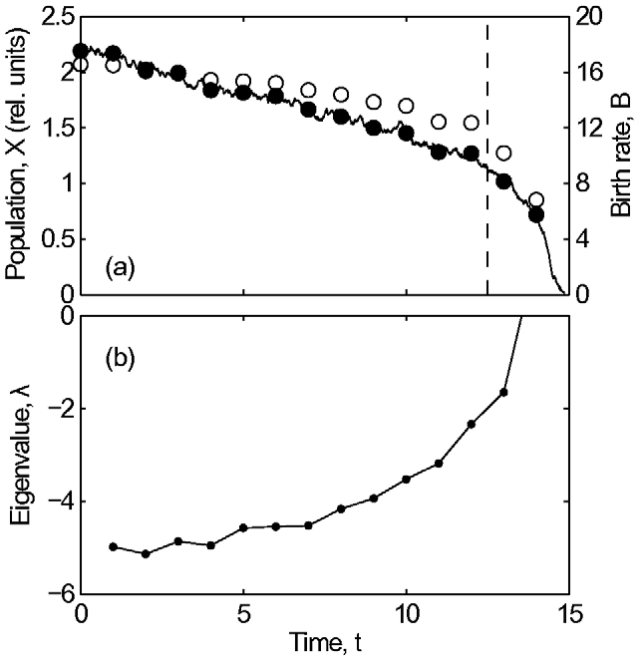
Early warning signal for a single population with Allee effect. (a) Population time series (solid line, left axis, in relative units) and yearly births (circles, right axis) generated by the simulation model described in Text S1. The vertical dashed line indicates the time of the bifurcation. Parameters were 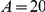, 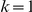, 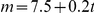 and 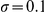. The simulation was started at 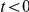 to allow for the decay of transient responses. (b) Eigenvalues estimated from the sampled data indicated with markers in (a), using the procedure described in the text. The eigenvalue was always real.

To test the early warning signal, we simulated a simple model (given in the Supporting Information as Text S1) of an Allee effect with additive noise. A critical transition occurred, causing subsequent extinction of the population (Fig. 2). The challenge we addressed is predicting the critical transition from a limited number (here, fifteen) of observations of population size and birth rate. We emphasize that we did not utilize any information on the functional forms of processes employed in the simulation, so that the prediction is based solely on the 15 observations and the assumed structural information (that is, one population subject to gains and losses). By estimating the parameters of the generalized model as described above, we determined the eigenvalues of the Jacobian as a function of time (Fig. 2b). A clear increase in the eigenvalue is detectable well before the critical transition, giving ample warning of the impending collapse.

Due to a phenomenon called bifurcation delay [23], the population size did not start to change rapidly until well after (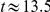) the bifurcation point (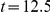). As previously observed by Biggs *et al.* [27], management action to reverse the change in bifurcation parameter may successfully avert the critical transition even after the fast subsystem's bifurcation has occurred, if still within the range of the bifurcation delay. In the case of Fig. 2b, the eigenvalue trend is directed more towards the last possible time that successful management action can be taken than towards the time of the actual bifurcation.

### Fishery simulation of Biggs *et al.*

Our second case study focuses on an example from fisheries. Increased harvesting of piscivores can induce a shift from the high-piscivore low-planktivore regime to a low-piscivore high-planktivore regime [28]. Many fisheries are suspected to have undergone such transitions [29], [30]. Based on the causal loop diagram (Fig. 1), we formulated a discrete-time generalized model, describing the piscivore and planktivore populations at the end of each year, in the spirit of stock-assessment modeling (see Text S1). Thereby detailed modeling of the intra-annual dynamics was avoided.

**Figure 3:**
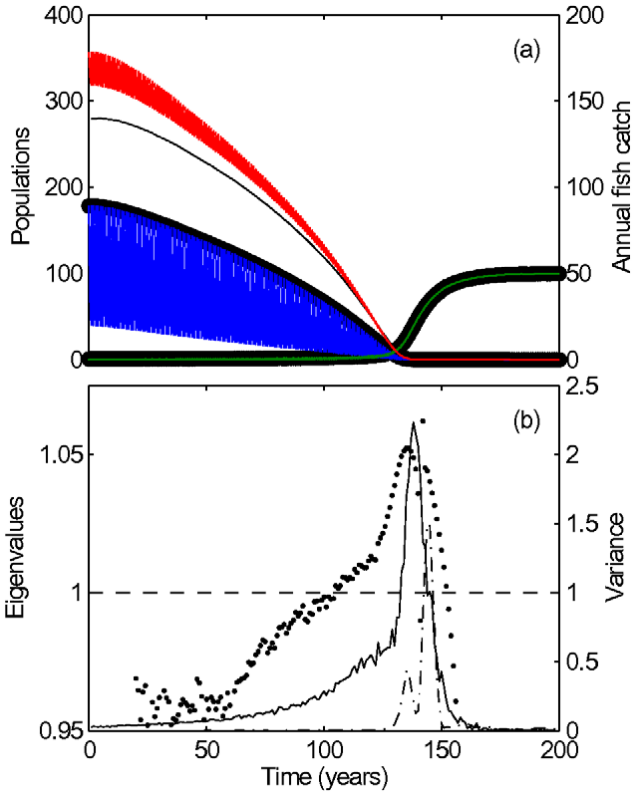
Early warning signals for the fishery simulation of Biggs *et al.* [**27**].

To test the warning signal, we generated time series data with a detailed fishery model by Biggs *et al.* [27], which was developed from a series of whole-lake experiments [31]. We describe this model more fully in Text S1, but note here that the model differs significantly from our generalized model by a) accounting for the intra-annual dynamics and b) containing an additional state variable denoting the juvenile piscivore population. These discrepancies were intentionally included to reflect the limited information that would be available for the formulation of the generalized model in practice. In simulations the detailed model showed a transition to a low-piscivore high-planktivore regime as the harvesting rate was increased (Fig. 3a).

From this simulation, we recorded the simulated piscivore and planktivore density and piscivore catch at the end of each year. Because the simulated data was very noisy we estimated the Jacobian's eigenvalues after smoothing the recorded data (see Text S1). In addition to the time series data, the information on the natural adult piscivore mortality and reproduction rate and the planktivore influx from refugia were required (see Text S1). This type of information can be reasonably well estimated for most systems. We confirmed that our predictions (reported below) are not sensitive to the specific values used. Indeed, a simple approach for estimating these parameters is to recognize that the initial state, before the critical transition, is stable. In a number of test trials we confirmed that any reasonable combination of parameters used that corresponded to an initially stable steady state provided an early warning signal comparable to the results reported below.

An estimate of the Jacobian eigenvalues for the fisheries example is shown in Fig. 3b. As the system approaches the critical transition we observe that an eigenvalue approaches one, which signifies a critical transition for discrete time systems. This result is compared to the variance early warning signal computed by Biggs *et al.* [27], which uses a much more densely sampled time series including intra-annual dynamics. The comparison shows that the approach proposed here produces a signal of similar quality (although possibly *too* early), while requiring significantly less time series data. Further, comparison with a variance signal using the same amount of time-series data as the generalized model shows that the generalized model-based signal is a much clearer early warning signal in this case. In particular, the variance signal only rises during or after the transition.

### Tri-trophic food chain

For our final example we consider a tri-trophic food chain. In ecological theory food chains play a role both as a prominent motif appearing in complex food webs and as coarse-grained models. Using generalized models, a general Jacobian for a continuous-time model of the tri-trophic food chain can be derived (see Text S1 and Gross *et al.* [32]).

**Figure 4:**
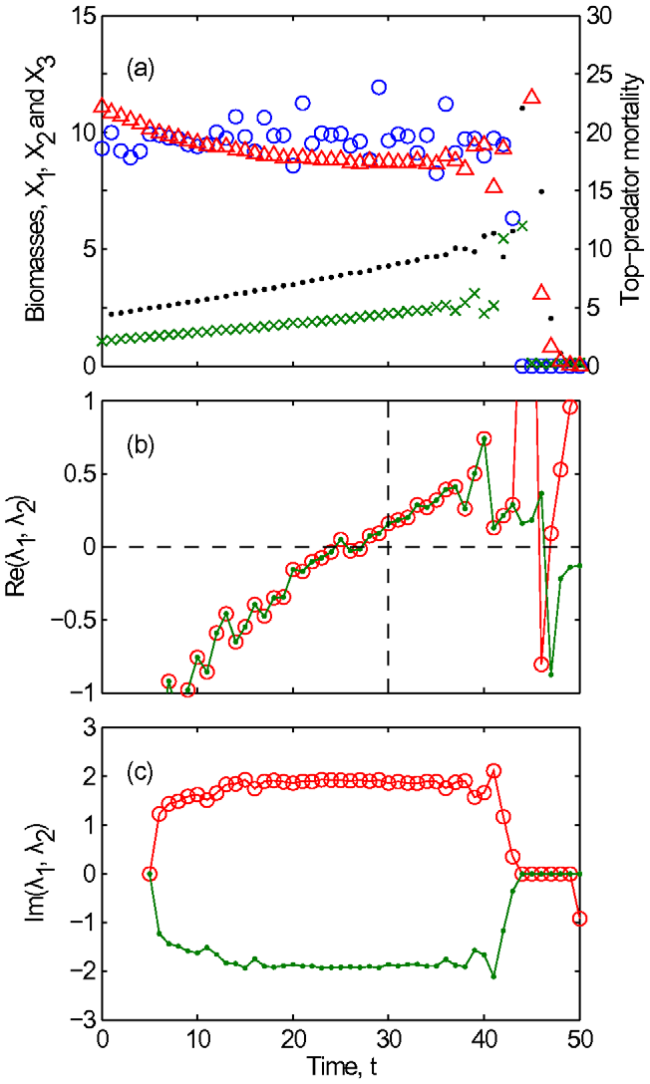
Early warning signal for a critical transition in a tri-trophic food chain. (a) Time series of 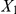 (blue circles, left axis), 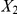 (green crosses, left axis) and 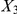 (red triangles, left axis) generated by the simulation model described in Text S1. Only the data subsequently used in the early warning analysis were plotted. Some of the data during the oscillations between 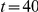 and 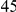 were outside the scale of this graph, with 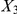 exceeding 30. The estimates of top-predator mortality used to calculate the early warning signal are also shown (black dots, right axis). Parameters were 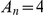, 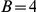, 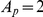, 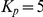, 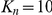, 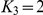, 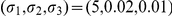 and 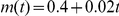. (b,c) Real and imaginary parts of eigenvalues estimated from the data in (a), using the procedure described in the text. Eigenvalues are denoted by dots and circles; a dot within a circle indicates two eigenvalues had the same real value. The markers and colors used in (b,c) have no correspondence to those used in (a). A third (purely real) eigenvalue was not plotted, for reasons described in the main text. In (b), the horizontal dashed line indicates the stability boundary at zero real part, while the vertical dashed line indicates the time that the (Hopf) bifurcation occurred in the fast subsystem of the simulation model.

We generated example time series data using a set of three ordinary differential equations that modeled a producer biomass, 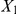, predator biomass, 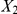, and top predator biomass, 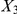, as described in Text S1. We included additive noise terms in the equations, and if any biomass decreased to zero we suppressed the noise term so that the corresponding population remained extinct. We simulated these equations while increasing the mortality rate 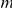 of the top predator. The resulting time series, for the chosen combination of parameters, show a slowly changing steady-state followed by a sudden transition to large oscillations, and a sudden collapse of all three populations (Fig. 4).

To provide an early warning signal for the transition we recorded time series of the three biomasses and the top-predator's death rate, and estimated the parameters of the generalized model from smoothed segments of these time series. Even for the smoothed data we find that one of the eigenvalues is very noisy and sometimes positive. We believe that the presence of this eigenvalue reflects the response of the prey to fluctuations on the higher trophic levels and therefore exclude this value from our analysis. As 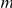 is increased toward the onset of oscillations, two eigenvalues show a clear increase toward zero real part (Fig. 4). The two eigenvalues approach zero as a complex conjugate eigenvalue pair, which is indicative of the system undergoing a Hopf bifurcation [24], which in turn generally implies a transition from stationary to oscillatory dynamics. The early warning signal constructed here, consisting of the approach of this eigenvalue pair towards the imaginary axis, warned of the transition to an oscillatory state significantly before the transition occurred. These large oscillations combined with stochastic fluctuations then led rapidly to extinction.

Supercritical Hopf bifurcations, to which class the bifurcation in the present system belongs, are by themselves not critical transitions. The detection of Hopf bifurcations is nevertheless of interest. First, subcritical Hopf bifurcations are indeed true critical transitions. Second, even supercritical Hopf bifurcations have long been associated in ecology with rapid destabilization and extinction of populations [33], a chain of events that we characterize as a critical transition and that we observed to occur in the present system. We also note that although to linear order sub- and super-critical Hopf bifurcations cannot be distinguished, generalized modeling can be extended to higher orders where these cases can be identified [34].

## Discussion

In this Letter we proposed an approach for anticipating critical transitions before they occur. In particular we showed that generalized modeling of the system can facilitate the incorporation of the structural information that is in general available.

We demonstrated the proposed approach in a series of three case studies. The first example showed that in simple systems even very few time points can be sufficient for clean prediction of the critical transition. The second example posed a hard challenge, where test data was generated by a model that differed considerably from the generalized model. Yet even in this case the generalized model significantly reduced the amount of data needed for predicting the transition. The third and final example demonstrated the ability of the proposed approach to distinguish between different types of critical transitions (in this case, through the presence of a complex conjugate pair of eigenvalues approaching the imaginary axis).

In all case studies we found that the proposed approach can robustly warn of critical transitions in the presence of noise. We believe that the performance of the approach under noisy conditions can be further improved by subsequent refinements. Such refinements could include combination with dynamic linear modeling [14], utilization of a parameter transformation (to ‘scale’ and ‘elasticity’ parameters) previously proposed for generalized models [20], or the use of optimized sampling procedures.

Two important rules for constructing the generalized model are as follows. First, there must be sufficiently few unobservable processes (represented by placeholder functions in the generalized model) that their magnitudes can be inferred from balancing the observable processes. For example, in the Allee effect study, the unobserved process 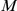 was estimated by 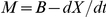. Second, where a process is a function of 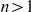 variables (although in the cases studied here 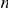 was never larger than 2), our method at present requires assumptions or other knowledge about the dependence of the process on 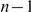 of those variables. This requirement could be relaxed in future work, although probably at the cost of requiring more time series data.

An advantage of the proposed approach is that it generally becomes more reliable closer to a critical transition, where rates of change of state variables and other observables are generally larger, which may lead to better sampling, although noise will also increase close to the transition due to critical slowing down. In such situations statistical methods such as variance may become more difficult to estimate as the time series becomes less stationary, for example since detrending becomes more difficult. On the other hand, the model-free statistical approaches may be more useful where little knowledge is available about the system, or where trends in the means of observed quantities are strongly obscured by noise. In these respects, the proposed approach provides a tool complementary to established statistical methods, each method with its own domain of utility.

One limitation shared by both our and the statistical early warning methods is that large noise can bias the estimation of the respective warning signals. In our case, an asymmetric distribution of fluctuations can bias the estimation of the underlying steady state. That our approach effectively involves derivatives of time series can increase the sensitivity to high observation noise or otherwise poor-quality data. Another important assumption in our present treatment (that is also shared by the statistical approaches) was that the dynamics of the fast subsystem could, at least at some level of description, be considered as stationary. Let us emphasize that this is not a strong assumption because even systems that are primarily non-stationary, such as the fisheries example, can be modeled as stationary if a suitable generalized modeling framework is chosen. Furthermore, ongoing efforts aim at extending the framework of generalized modeling to non-stationary dynamics, which may lead to a further relaxation of that assumption in the future [35].

A thorough statistical analysis of the generalized modeling and the statistics-based approaches is another topic for future work. Such a study would help to quantify under exactly what conditions the generalized modeling approach can operate effectively and offer advantages compared to statistics-based approaches.

In summary, we used generalized models to efficiently incorporate available information about a system without requiring detailed knowledge about that system. Our intermediate-complexity method provides an early warning signal approach complementary to existing statistics-based methods. In the cases studied here, our method could provide early warning signals with significantly less time series data than statistical approaches. Thereby the proposed approach can, under suitable conditions and with good quality data, contribute to the warning of critical transitions from a realistic sampling effort.

## Supporting Information

Text S1 **Simulation models and detailed method for early warning signal calculation.** Text S1 details the application of the generalized modeling-based early warning approach to the fishery simulation and the tri-trophic food chain simulation. (The method for the Allee effect application is described in the main text.) The simulation models, used to generate the data to which the early warning signals were applied, are also described.
(PDF)
