# Supplementary material for: Early Warning Signals for Critical Transitions: A Generalized Modeling Approach
Source: PLoS Comput Biol. 2012 Feb 2;8(2):e1002360. doi: 10.1371/journal.pcbi.1002360 (PMC3271022; doi:10.1371/journal.pcbi.1002360)
Supplement: Text S1 — Simulation models and detailed method for early warning signal calculation. Text S1 details the application of the generalized modeling-based early warning approach to the fishery simulation and the tri-trophic food chain simulation. (The method for the Allee effect application is described in the main text.) The simulation models, used to generate the data to which the early warning signals were applied, are also described. (PDF) [file pcbi.1002360.s001.pdf]

Text S1: Simulation models and detailed method for early warning signal calculation

Steven J. Lade*, Thilo Gross
Max Planck Institute for the Physics of Complex Systems, Nöthnitzer Str. 38, 01187 Dresden, Germany
∗ E-mail: slade@pks.mpg.de

This Supporting Text details the application of the generalized modeling-based early warning approach to the fishery simulation and the tri-trophic food chain simulation. (The method for the Allee effect application is described in the main text.) The simulation models, used to generate the data to which the early warning signals were applied, are also described.

Early warning signal calculation

Fishery simulation of Biggs et al.

We assumed the following knowledge:

- The populations of adult piscivores A_i and planktivores F_i at the start of year i
- The number of adult piscivores harvested $Q_i = Q(A_i)$ during year i
- That adult piscivores die from other causes at a per-capita rate m
- That adult piscivores predate on the planktivores
- That there is a net movement of planktivores into (out of) the foraging arenas from (to) refugia [2] of R_i in year i
- That per-capita, and in the absence of interactions with other modeled populations, r piscivores would be born and mature into adult piscivores the following year
- That however juvenile piscivores are predated on by planktivores [3] and are also controlled, for example by accidental predation, by the adult piscivores [4].

This knowledge is represented schematically in Fig. 1 of the main text.

We constructed a generalized model of this fishery by modeling only the year-end piscivore populations A_i and planktivore populations F_i with a discrete map. As a consequence we could avoid explicitly modeling the complications of intra-annual dynamics such as piscivore reproduction and maturation. We wrote:

$$A_{i+1} = (1-m)(A_i - Q_i) + rA_i - C_i \tag{1a}$$

$$F_{i+1} = F_i + R_i - D_i. \tag{1b}$$

The first term of Eq. (1a) gives the number of surviving adult piscivores after harvesting (Q_i) and other causes of mortality (m). The second term rA_i models reproduction without losses and the third $C_i = C(A_i, F_i)$ gives the predatory or controlling effects of planktivores and adult piscivores on the number of new adult piscivores. Since C depends twice on the adult piscivore population, first through the number of juvenile piscivores born and again through the controlling effect of the adults, we assume C is quadratic in A_i.

In Eq. (1b), R_i models the net influx of planktivores into the foraging arenas. Since the planktivore population was initially small, we assumed there was negligible outflow of planktivores, and that the inflow depended on a refuge size that was constant. Therefore $R_i = R$ was constant. [In the other

scenario considered by Biggs *et al.* [5], where habitat destruction causes the refuge size to decrease, R_i would not be constant.] The remaining term $D_i = D(A_i, F_i)$ models all other factors changing the planktivore population, most importantly predation by adult piscivores. Since the planktivore population was initially small, we assumed D to be linear in F_i.

From the output of the simulation model (described below) we recorded annual adult piscivore density, planktivore density, and piscivore catch. Since the ecosystem approached the critical transition more slowly than the model with Allee effect above, to remove noise we found it necessary to smooth estimates of the gradients. For each new observation at time t_i, we fitted a second-order polynomial (since the method eventually results in differences of up to second order) to the record of each observed time series up to that time. Since it is the gradients at the current time that are most important, in the fit we applied a weight to each data point j of $\exp[(t_j - t_i)/\tau]$ with $\tau = 10$. Gradients were then extracted directly from the coefficients of the polynomial fit. This simulated the calculation of early warning signals 'on the fly', as new observations become available.

Specifically, at each time i we fitted the three polynomials

$$\begin{aligned}
\tilde{F}_j &= p_{F0} + p_{F1}(j-i-1) + p_{F2}(j-i-1)^2, \quad & j &= 0, \ldots, i+1 \\
\tilde{A}_j &= p_{A0} + p_{A1}(j-i-1) + p_{A2}(j-i-1)^2, \quad & j &= 0, \ldots, i+1 \\
\tilde{Q}_j &= p_{Q0} + p_{Q1}(j-i) + p_{Q2}(j-i)^2, & j &= 0, \ldots, i
\end{aligned}$$

to our three input time series. We assume that at year i, as well as the fish harvest for that year (Q_i) we also have already available the piscivore and planktivore populations at the end of that year (A_{i+1} and F_{i+1}, respectively). Changing this assumption and using only previous population observations does not significantly change our results.

These smoothed observations were sufficient to calculate the Jacobian of Eqs. (1),

$$J = \begin{bmatrix} 1 - m + r - (1-m)Q'^{(A)} - C'^{(A)} & -C'^{(F)} \\ -D'^{(A)} & 1 - D'^{(F)} \end{bmatrix},$$

where we use the notation $\partial H/\partial x \equiv H'^{(x)}$. The eigenvalues λ of the Jacobian, which may be complex numbers, are the solutions of $\det(J - \lambda I) = 0$ where I is the (2×2) identity matrix.

Our procedure to estimate the Jacobian at time i was then as follows. From the assumption of linearity of Q in A,

$$\tilde{Q}'^{(A)} = \tilde{Q}/\tilde{A} = p_{Q0}/p_{A0}.$$

Estimates of $\tilde{C}$ and $\tilde{D}$ can be found by solving Eqs. (1). Together with the assumptions that C is quadratic in A and D linear in F, it follows that

$$\begin{aligned}
\tilde{C}'^{(A)} &= 2\tilde{C}/\tilde{A} = 2\left(-p_{A0} + (1+r-m)\tilde{A}_i - (1-m)p_{Q0}\right)/p_{A0} \\
\tilde{D}'^{(F)} &= \tilde{D}/\tilde{F} = \left(-p_{F1} + R\right)/p_{F0}
\end{aligned}$$

Finally, by solving linear Taylor expansions of the form

$$\Delta C = C'^{(A)}\Delta A + C'^{(F)}\Delta F, \tag{2}$$

the derivatives

$$\begin{aligned}
\tilde{C}'^{(F)} &= \frac{\Delta C - \tilde{C}'^{(A)}\Delta A}{\Delta F} = \left(-p_{A0} + (r-m)\tilde{A}_i - (1+r-m)\tilde{A}_{i-1} - (1-m)p_{Q1} - \tilde{C}'^{(A)}p_{A1}\right)/p_{F1} \\
\tilde{D}'^{(A)} &= \frac{\Delta D - \tilde{D}'^{(F)}\Delta F}{\Delta A} = \left(-2p_{F2} - \tilde{D}'^{(F)}p_{F1}\right)/p_{A1}
\end{aligned}$$

were estimated.

This generalized modeling approach required estimates of the adult piscivore mortality m and reproduction rate r, and planktivore influx R from refugia. We assumed the fishery manager could estimate $m = 0.4$ and $r = 1.1$ from knowledge of the fish species and the fishery. Effective values $m = 0.5$ and $r = 1$ can be derived from the actual simulation's equations [5]. The planktivore influx R would be harder to estimate in practice. In the absence of such knowledge, the ecosystem manager could make use of the fact that the fish populations were initially stable. This knowledge constrains R to the range 0.1 to 1.5, as values outside these ranges produce an eigenvalue with magnitude greater than one between times $t = 0$ to 10. We used $R = 0.9$. Indeed, this knowledge could also be used to constrain m and r to the ranges 0.1 to 0.7 and 0.95 to 1.5, respectively. We found that any combination of m, r and R that gave an initially stable system robustly predicted the later critical transition.

Tri-trophic food chain

To obtain an early warning signal for this transition, we constructed a generalized model of the tri-trophic food chain

$$\frac{\mathrm{d}}{\mathrm{d}t}X_1 = A(X_1) - G(X_1, X_2) \tag{3a}$$

$$\frac{\mathrm{d}}{\mathrm{d}t}X_2 = G(X_1, X_2) - H(X_2, X_3) \tag{3b}$$

$$\frac{\mathrm{d}}{\mathrm{d}t}X_3 = H(X_2, X_3) - M(X_3, \mu), \tag{3c}$$

where X_1, X_2 and X_3 are the biomasses of the three species in the food chain, $A(X_1)$ is the primary production rate of X_1, $G(X_1, X_2)$ and $H(X_2, X_3)$ are the rates at which X_2 and X_3 consume X_1 and X_2, respectively, and $M(X_3, \mu)$ is the total mortality per unit time of the top-level predator X_3, with the argument μ denoting it is subject to a changing external influence. The approach could also easily be extended to the case of incomplete biomass conversion efficiencies, if estimates of those efficiencies were available.

We assumed: access to observations of all three biomasses X_1, X_2 and X_3, and the top-predator mortality $M(X_3, \mu)$; that this mortality is linear in X_3 so that $M = m(\mu)X_3$; and that the predation rates $G(X_1, X_2)$ and $H(X_2, X_3)$ are linear in their respective predator biomasses. The 'observed' time series were first smoothed by the same filter as in the fishery simulation, but with $\tau = 20$.

The Jacobian matrix of Eqs. (3) is

$$J = \begin{bmatrix} A'^{(1)} - G'^{(1)} & -G'^{(2)} & 0 \\ G'^{(1)} & G'^{(2)} - H'^{(2)} & -H'^{(3)} \\ 0 & H'^{(2)} & H'^{(3)} - M'^{(3)} \end{bmatrix},$$

where we denote $F'^{(a)} \equiv \partial F / \partial X_a$. We computed the quantities in the Jacobian as follows. By rearranging Eqs. (3), first H then G then A can be found. With the assumptions of linearity listed above, it follows that

$$\begin{aligned} \tilde{M}'^{(3)} &= \tilde{M}/\tilde{X}_3 = p_{M0}/p_{X_3,0} \\ \tilde{H}'^{(3)} &= \tilde{H}/\tilde{X}_3 = \left(p_{X_3,1} + p_{M0}\right)/p_{X_3,0} \\ \tilde{G}'^{(2)} &= \tilde{G}/\tilde{X}_2 = \left(p_{X_2,1} + p_{X_3,1} + p_{M0}\right)/p_{X_2,0}. \end{aligned}$$

Through a Taylor expansion as in Eq. (2),

$$\begin{aligned} \tilde{H}'^{(2)} &= \left(\Delta H - \tilde{H}'^{(3)}\Delta X_3\right)/\Delta X_2 = \left(2p_{X_3,2} + p_{M1} - \tilde{H}'^{(3)}p_{X_3,1}\right)/p_{X_2,1} \\ \tilde{G}'^{(1)} &= \left(\Delta G - \tilde{G}'^{(2)}\Delta X_2\right)/\Delta X_1 = \left(2p_{X_2,2} + 2p_{X_3,2} + p_{M1} - \tilde{G}'^{(2)}p_{X_2,1}\right)/p_{X_1,1} \end{aligned}$$

The last unknown in the Jacobian to be determined is

$$\tilde{A}'^{(1)} = \left(2p_{X_1,2} + 2p_{X_2,2} + 2p_{X_3,2} + p_{M1}\right)/p_{X_1,1}.$$

Simulation models

Single population with Allee effect

To test the early warning signal described in the main text we simulated a simple model

$$\frac{\mathrm{d}}{\mathrm{dt}}X = \frac{AX^2}{k^2 + X^2} - mX + \sigma\xi(t), \tag{4}$$

based on Yeakel *et al.* [1]. We included a small additive noise term $\sigma\xi(t)$, with standard deviation σ and autocorrelation $\langle\xi(t)\xi(t')\rangle = \delta(t - t')$. Throughout the simulation we slowly changed the mortality rate m according to $m = 7.5 + 0.2t$.

Fishery simulation of Biggs et al.

We generated data for this early warning signal test with the previously published fishery model of Biggs *et al.* [5]. Their model is a hybrid discrete-continuous system and explicitly models the adult piscivore, juvenile piscivore and planktivore populations. The intra-annual dynamics are continuous, modeling: the harvest of adult piscivores; the predation and control of planktivores and juvenile piscivores, respectively, by adult piscivores; predation on juvenile piscivores by planktivores; and the movement of both planktivores and juvenile piscivores between the foraging arenas and refugia. At the end of each year discrete update rules were applied that controlled the mortality of adult piscivores, maturation of the surviving juvenile piscivores into adult piscivores, and birth of more juvenile piscivores. There was an additive noise term on the planktivore population dynamics. For further details of the model including its mathematical formulation see Biggs *et al.* [5].

Tri-trophic food chain

To generate example time series data we used a conventional equation-based model in which a producer biomass, X_1, predator biomass, X_2, and top predator biomass, X_3, follow

$$\frac{\mathrm{d}}{\mathrm{dt}}X_1 = A_n X_1(K_n - X_1) - \frac{BX_1^2X_2}{K_3 + X_1^2} + \sigma_1\xi_1(t) \tag{5a}$$

$$\frac{\mathrm{d}}{\mathrm{dt}}X_2 = \frac{BX_1^2X_2}{K_3 + X_1^2} - \frac{A_pX_2^2X_3}{K_p + X_2^2} + \sigma_2\xi_2(t) \tag{5b}$$

$$\frac{\mathrm{d}}{\mathrm{dt}}X_3 = \frac{A_pX_2^2X_3}{K_p + X_2^2} - mX_3 + \sigma_3\xi_3(t). \tag{5c}$$

In these equations we assumed Holling type-3 predator-prey interaction, logistic growth of the producer and linear mortality of the top predator and $\xi_i(t)$ are additive noise terms with $\langle\xi_i(t)\xi_j(t')\rangle = \delta_{ij}\delta(t-t')$. If any biomass decreased to zero, we suppressed the noise term so that the corresponding population remained extinct.

References

1. Yeakel JD, Stiefs D, Novak M, Gross T (2011) Generalized modeling of ecological population dynamics. Theor Ecol 4: 179–194.

2. Walters CJ, Martell SJD (2004) Fisheries Ecology and Management. Princeton University Press.

3. Walters C, Kitchell JF (2001) Cultivation/depensation effects on juvenile survival and recruitment: Implications for the theory of fishing. Can J Fish Aquat Sci 58: 39–50.

4. Carpenter SR, Brock WA, Cole JJ, Kitchell JF, Pace ML (2008) Leading indicators of trophic cascades. Ecol Lett 11: 128–138.

5. Biggs R, Carpenter SR, Brock WA (2009) Turning back from the brink: Detecting an impending regime shift in time to avert it. Proc Natl Acad Sci USA 106: 826-831.
